# Inequality and determinants of access to phototherapy among patients with psoriasis in Thailand

**DOI:** 10.1093/skinhd/vzag110

**Published:** 2026-07-22

**Authors:** Chutima Churee, Piya Hanvoravongchai, Thanapoom Rattananupong

**Affiliations:** Photodermatology Division, Institute of Dermatology, Department of Medical Services, Ministry of Public Health, Bangkok, Thailand; Department of Preventive and Social Medicine, Faculty of Medicine, Chulalongkorn University, Bangkok, Thailand; Department of Preventive and Social Medicine, Faculty of Medicine, Chulalongkorn University, Bangkok, Thailand; Saw Swee Hock School of Public Health, National University of Singapore, Singapore,Singapore; Department of Preventive and Social Medicine, Faculty of Medicine, Chulalongkorn University, Bangkok, Thailand

## Abstract

**Background:**

Ultraviolet (UV) phototherapy is a cost-effective, guideline-recommended treatment for moderate-to-severe psoriasis, supported by extensive evidence demonstrating its long-term safety and clinical efficacy. As a foundational therapeutic option in routine dermatological practice, access to phototherapy contributes meaningfully to sustained disease control and overall patient outcomes. Nevertheless, comprehensive nationwide data describing real-world utilization, particularly within universal health coverage systems, remain limited. A clearer understanding of patterns of access may therefore offer valuable insight into the organization and delivery of dermatological care.

**Objectives:**

To quantify socioeconomic and regional inequality in UV phototherapy utilization and identify determinants of access within a universal health coverage system.

**Methods:**

This nationwide cross-sectional study (January–December 2023) integrated individual-level administrative health insurance data with a facility-based survey conducted across Thailand’s 13 health regions to assess the availability of dermatologists and UV phototherapy devices. Patients with psoriasis (*N* = 49 219) were identified using International Classification of Diseases (ICD)-10 codes L40.0–L40.9. Moderate-to-severe disease (*n* = 16 188) was defined using treatment-based proxies, including receipt of phototherapy, systemic agents or biologic therapies identified through ICD-9 procedure codes and Thai Medicine Terminology drug codes. The primary outcome was phototherapy utilization. Multivariable logistic regression was applied to estimate adjusted odds ratios. Socioeconomic inequality was quantified using the Concentration Index and concentration curves.

**Results:**

Among 49 219 identified patients with psoriasis (202 776 encounters), 16 188 were classified as having moderate-to-­severe disease. Of these, only 3.3% received UV phototherapy. Utilization was concentrated in higher-income provinces, as reflected by a positive Concentration Index of 0.213. In multivariable analysis, access to phototherapy was independently associated with age, psoriasis subtype, insurance scheme, geographical region, provincial income level, and the availability of dermatologists and phototherapy devices.

**Conclusions:**

Access to phototherapy in Thailand remains limited and unequally distributed despite universal insurance coverage. Reducing disparities in workforce distribution and service capacity may enhance equitable access to this core dermatological treatment modality and support more equitable long-term disease control in routine clinical practice.

## Teaser

This nationwide analytical cross-sectional study examined inequality in access to phototherapy among patients with psoriasis in Thailand using national insurance databases and a hospital facility survey. Only 3.3% of patients received phototherapy, with pro-rich inequality (Concentration Index = 0.213). Socioeconomic and workforce disparities, particularly unequal dermatologist distribution, remain key barriers to equitable access.

What is already known about this topic?

- Ultraviolet (UV) phototherapy is a guideline-endorsed, evidence-based and cost-effective treatment for moderate-to-severe psoriasis.
- Despite universal health coverage ensuring financial protection in Thailand, phototherapy units and utilization remain limited, especially outside urban centres.
- Socioeconomic and regional inequities are known to influence dermatological care access, yet no nationwide evidence has assessed inequality in phototherapy utilization among patients with psoriasis.

What does this study add?

- This study provides the first nationwide evidence quantifying socioeconomic and regional inequality in access to phototherapy for psoriasis in Thailand, integrating patient-level insurance data with a facility survey across all 13 health regions.
- Only 3.3% of patients with moderate-to-severe psoriasis received UV phototherapy (Concentration Index = 0.213), indicating a strong pro-wealthy distribution.
- Findings reveal structural inequities in dermatology workforce and phototherapy resources, underscoring the urgent need for equity-oriented and regionally targeted health resource allocation.

Psoriasis is a chronic immune-mediated inflammatory disease that imposes a substantial and enduring burden on patients, encompassing physical discomfort, visible disfigurement, psychosocial distress and impaired quality of life.^1–3^ In patients with ­moderate-to-severe disease, phototherapy, particularly narrowband ultraviolet B (NB-UVB), remains a cornerstone of management owing to its well-established efficacy, favourable long-term safety profile and cost-effectiveness relative to many systemic agents.^4,5^

Timely access to appropriate treatment is essential for sustained disease control and mitigation of cumulative inflammatory burden. Delayed or restricted access to guideline-recommended therapies may result in persistent disease activity and increased risk of psoriatic arthritis and cardiometabolic comorbidities.^6–8^ Ensuring the availability of effective therapeutic modalities is therefore fundamental to optimal long-term clinical outcomes.

Despite its established role in dermatological practice, access to phototherapy is not uniformly distributed. Utilization has been shown to vary according to geographical location, travel distance, insurance coverage, and the availability of dermatology specialists and phototherapy facilities.^9–11^ Workforce maldistribution and infrastructure constraints may limit delivery of specialist-based treatments, particularly in resource-constrained settings.^12,13^

Although disparities in psoriasis care have been described in several high-income countries, most evidence derives from regional or single-centre analyses. Nationwide evaluations integrating individual-level utilization data with structural healthcare factors remain limited, especially in middle-income countries operating under universal health coverage systems.

Thailand provides a compelling context for examining these issues, as NB-UVB phototherapy is integrated into its universal health coverage framework. Nevertheless, financial protection alone may not guarantee equitable real-world access if specialist distribution and facility capacity remain geographically uneven.

Accordingly, this nationwide cross-sectional study aimed to evaluate patterns of phototherapy utilization among patients with psoriasis in Thailand and to identify clinical and health system determinants associated with access to treatment. By integrating administrative utilization data with facility-level information, the study sought to provide clinically relevant evidence to inform equitable delivery of phototherapy within routine dermatological practice.

## Materials and methods

### Data sources and data collection

This nationwide cross-sectional study was conducted between January and December 2023 within Thailand’s universal health coverage system. Two data sources were integrated. Firstly, individual-level healthcare utilization data were obtained from national administrative databases covering the three major public insurance schemes: the National Health Security Office (NHSO), the Social Security Office (SSO), and the Central Office for Healthcare Information (CHI). Patients with psoriasis were identified using International Classification of Diseases (ICD)-10 codes L40.0–L40.9, requiring at least one outpatient or inpatient encounter during the study period. Records with incomplete treatment data were excluded.

A total of 202 776 encounters representing 49 219 unique individuals were identified. Among these, 16 188 patients with moderate-to-severe psoriasis comprised the analytic sample for multivariable and inequality analyses.

Secondly, a nationwide facility-level survey was conducted across all eligible regional and general hospitals affiliated with the Ministry of Public Health, including hospitals in all 13 health regions. Structured questionnaires collected information on the availability and type of UV phototherapy devices and the presence of dermatologists. Response rates exceeded 80%, with complete data obtained following follow-up.

### Patient classification and variables

Disease severity was classified using treatment-based proxies. Mild psoriasis was defined as receipt of topical therapy only, whereas moderate-to-severe psoriasis included patients receiving phototherapy, systemic agents or biologics, identified through ICD-9 procedure codes and Thai Medicine Terminology drug codes.

The primary outcome was utilization of UV phototherapy, defined as at least one documented session in the administrative records between January and December 2023.

Patient-level covariates included:

- Demographics: age group and sex
- Disease characteristics: psoriasis subtype (ICD-10 L40.0–L40.9) and treatment-based severity
- Health insurance scheme: Universal Coverage Scheme (UCS), Social Security Scheme (SSS), Civil Servant Medical Benefit Scheme (CSMBS)
- Geographical region: Bangkok, Central, Northern, Northeastern and Southern regions
- Socioeconomic status: five provincial income levels based on average monthly household income per capita (Thai Baht), as ­individual-level income and education data were unavailable

Facility-level covariates derived from the survey included health service availability, categorized as follows:

1. None = no dermatologist and no phototherapy device
2. Doctor only = dermatologist available but no phototherapy device
3. Derm and device = both dermatologist and phototherapy device available

### Statistical analysis

All eligible patients with psoriasis identified in the national healthcare utilization databases during 2023 were included; therefore, no a priori sample size calculation was required. Bivariate analyses were first conducted to examine associations between patient-level and contextual characteristics and phototherapy utilization. Variables were selected for multivariable modelling based on theo­retical frameworks, prior evidence and clinical relevance.

Multivariable logistic regression was applied to estimate adjusted odds ratios (aORs) with 95% confidence intervals (CIs). To assess potential effect modification, interaction terms between health insurance schemes and geographical regions were included in the models. Adjusted estimates were further explored using postestimation analyses to obtain region-specific effects. The statistical significance of interaction terms was assessed using Wald tests.

Robust standard errors accounted for heteroskedasticity and intraprovincial clustering. Model diagnostics included assessments of multicollinearity using correlation matrices and variance inflation factors, and model fit was evaluated using pseudo *R*^2^ and likelihood ratio tests.

Socioeconomic inequality in phototherapy access was quantified using the Concentration Index, calculated via a rank-­dependent method based on provincial per capita household income, and visualized using concentration curves. A positive Concen­tration Index indicated income-related concentration favouring higher-income provinces, whereas a negative Concen­tration Index reflected concentration among lower-income provinces.

All analyses were conducted using Stata version 14.1 (StataCorp., College Station, TX, USA). Statistical significance was defined as a two-sided *P*-value < 0.05.

## Results

### Characteristics of patients with psoriasis

In 2023, 49 219 individuals with physician-diagnosed psoriasis were identified, corresponding to 202 776 encounters (Table 1). The mean (SD) age of the individuals was 51.8 (15.6) years, and 56.7% (*n* = 27 915) were men. Over half (*n* = 28 221) were aged ≥50 years, with 32.8% (*n* = 16 148) aged ≥60 years. Overall, 32.9% (*n* = 16 188) were classified as having moderate-to-severe disease. Chronic plaque psoriasis was the most common subtype (*n* = 23 012; 46.8%).

**Table 1:** General characteristics of patients with mild-to-severe psoriasis in 2023 (*N* = 49 219)

Characteristic	*n* (%)
Sex	
Male	27 915 (56.7)
Female	21 304 (43.3)
Age (years)	
Mean (SD)	51.8 (15.6)
Range	1–103
Age group (years)	
≤19	1193 (2.4)
20–29	2788 (5.7)
30–39	6953 (14.1)
40–49	10 064 (20.4)
50–59	12 073 (24.5)
≥60	16 148 (32.8)
Severity of psoriasis	
Mild (topical therapy)	33 031 (67.1)
Moderate-to-severe (phototherapy, systemic agents or biologics)	16 188 (32.9)
Type of psoriasis	
Plaque psoriasis	23 012 (46.8)
Generalized pustular	515 (1.1)
Acrodermatitis continua	71 (0.1)
Palmoplantar pustulosis	136 (0.3)
Guttate psoriasis	569 (1.2)
Psoriatic arthritis	1762 (3.6)
Other specified	1295 (2.6)
Unspecified	21 859 (44.4)
Treatment received	
Topical therapy	33 031 (67.1)
Systemic therapy	15 559 (31.6)
Phototherapy	531 (1.1)
Biologic agents	98 (0.2)
Health insurance scheme	
SSS	21 396 (43.5)
CSMBS	14 151 (28.8)
UCS	13 672 (27.8)
Geographical region	
Bangkok	12 427 (25.2)
Central region	14 932 (30.3)
Northern region	6868 (14.0)
Northeastern region	10 482 (21.3)
Southern region	4510 (9.2)

Data are presented as *n* (%) unless otherwise stated. CSMBS, Civil Servant Medical Benefit Scheme; SSS, Social Security Scheme; UCS, Universal Coverage Scheme.

Most patients received topical therapy (*n* = 33 031; 67.1%), followed by systemic oral agents (*n* = 15 559; 31.6%), phototherapy (*n* = 531; 1.1%) and biologics (*n* = 98; 0.2%). By insurance scheme, 43.5% (*n* = 21 396) were covered by SSS, 28.8% (*n* = 14 151) by CSMBS and 27.8% (*n* = 13 672) by UCS. The largest proportions resided in the Central region (*n* = 14 932; 30.3%) and Bangkok (*n* = 12 427; 25.2%).

### Prevalence and geographical distribution

The overall age- and sex-adjusted prevalence of psoriasis in Thailand was 75.6 per 100 000 population in 2023, estimated from public-sector service utilization data and Thailand’s official mid-year population statistics using direct standardization. This figure may represent an underestimate of the true national prevalence, as private-sector data were not included. Prevalence increased with age, with the highest rates observed among individuals aged ≥60 years. Geographically, the Central and Northeastern regions accounted for the largest absolute number of cases, whereas Bangkok demonstrated the highest ­population-adjusted prevalence.

### Distribution of ultraviolet phototherapy resources

In 2023, 38 UV phototherapy devices were available in public facilities nationwide, comprising 13 whole-body NB-UVB devices, 12 localized NB-UVB devices, 8 whole-body UVA devices and 5 localized UVA devices. The distribution was markedly uneven: Bangkok had the highest number (*n* = 11), followed by the Southern region (*n* = 10), Central region (*n* = 9), Northeastern region (*n* = 5) and Northern region (*n* = 3). Device density per 100 000 patients with psoriasis varied substantially across regions, with the highest availability observed in the Southern and Bangkok, where the device-to-patient ratio was approximately 5.08 times higher than in the Northern (Figure 1).

**Figure 1:**
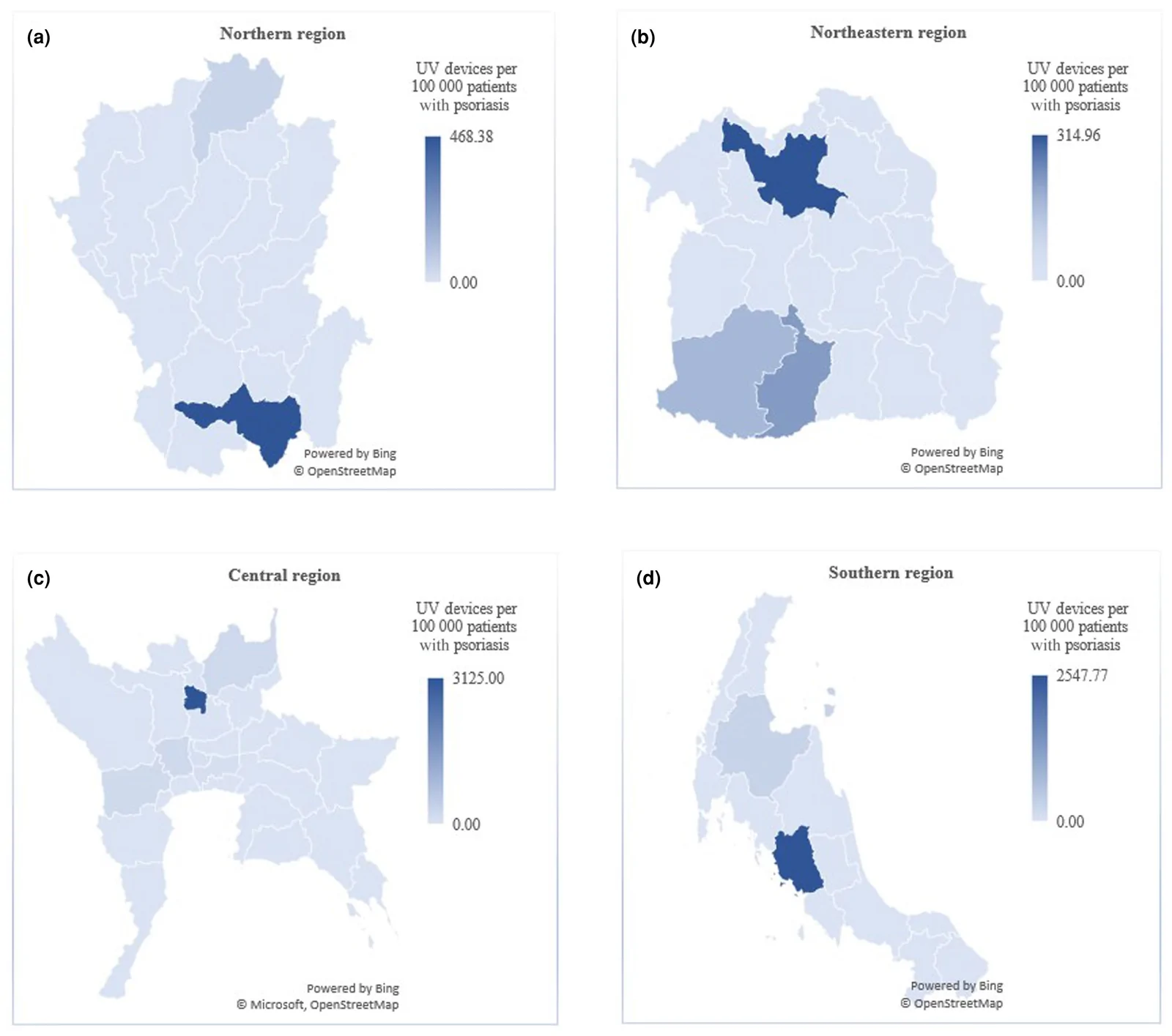
Geographical distribution of ultraviolet (UV) phototherapy devices across Thailand’s health regions, 2023. (a) Northern region. (b) Northeastern region. (c) Central region. (d) Southern region. Shading intensity indicates the number of UV devices per 100 000 patients with psoriasis, with darker shades reflecting higher device availability.

### Multivariable analysis of phototherapy access

Among 16 188 patients with moderate-to-severe psoriasis, 531 (3.3%) received phototherapy (Table 2). Compared with patients aged <50 years, those aged 50–59 years (aOR 0.58, 95% CI 0.44–0.76) and ≥60 years (aOR 0.62, 95% CI 0.48–0.80) had lower odds of treatment. Nonplaque subtypes were associated with a reduced likelihood of phototherapy (aOR 0.45, 95% CI 0.37–0.54).

**Table 2:** Factors associated with access to ultraviolet phototherapy among patients with moderate-to-severe psoriasis (*N* = 16 188)

Factor	Phototherapy access, *n* (%)		Crude OR (95% CI)	Adjusted OR (95% CI)
	Yes, *n* (row %) (*n* = 531)	No , *n* (row %) (*n* = 15 657)		
Age group (years)				
≤19	17 (5.3)	306 (94.7)	1.68 (1.00–2.82)	1.13 (0.65–1.94)
20–29	47 (4.9)	911 (95.1)	1.56 (1.10–2.20)*	1.34 (0.94–1.91)
30–39	69 (2.8)	2364 (97.2)	0.88 (0.65–1.19)	0.89 (0.65–1.21)
40–49	118 (3.2)	3562 (96.8)	1.00	1.00
50–59	106 (2.5)	4208 (97.5)	0.76 (0.58–0.99)*	0.58 (0.44–0.76)***
≥60	174 (3.9)	4306 (96.1)	1.22 (0.96–1.55)	0.62 (0.48–0.80)***
Type of psoriasis				
Plaque psoriasis	351 (5.3)	6309 (94.7)	1.00	1.00
Other psoriasis	180 (1.9)	9348 (98.1)	0.35 (0.29–0.42)***	0.45 (0.37–0.54)***
Health insurance scheme				
UCS	228 (3.6)	6145 (96.4)	1.00	1.00
CSMBS	191 (7.2)	2473 (92.8)	2.08 (1.71–2.54)***	1.88 (1.52–2.34)***
SSS	112 (1.6)	7039 (98.4)	0.43 (0.34–0.54)***	0.33 (0.26–0.43)***
Geographical region				
Bangkok	204 (6.4)	2965 (93.6)	1.00	1.00
Central region	101 (2.3)	4320 (97.7)	0.34 (0.27–0.43)***	0.54 (0.38–0.75)***
Northern region	70 (2.9)	2380 (97.1)	0.43 (0.32–0.56)***	0.81 (0.50–1.31)
Northeastern region	97 (2.2)	4321 (97.8)	0.33 (0.26–0.42)***	0.51 (0.33–0.79)**
Southern region	59 (3.4)	1671 (96.6)	0.51 (0.38–0.69)***	0.60 (0.42–0.85)**
Socioeconomic status (Thai Bhat)				
≤21 212	36 (1.2)	3006 (98.8)	0.96 (0.52–1.76)	0.84 (0.45–1.59)
21 213–24 039	40 (1.8)	2129 (98.2)	1.50 (0.83–2.73)	1.30 (0.71–2.40)
24 040–26 759	15 (1.2)	1200 (98.8)	1.00	1.00
26 760–31 368	144 (5.0)	2741 (95.0)	4.20 (2.46–7.18)***	3.57 (2.04–6.26)^***^
>31 368	296 (4.3)	6581 (95.7)	3.62 (2.13–6.07)***	2.13 (1.19–3.81)**
Health service availability				
None	20 (1.1)	1830 (98.9)	1.00	1.00
Doctor only	168 (2.1)	7783 (97.9)	1.97 (1.24–3.15)^**^	1.51 (0.92–2.48)
Derm & device	343 (5.4)	6044 (94.6)	5.19 (3.30–8.16)^***^	2.51 (1.51–4.16)^***^

Regression models were adjusted for age group, psoriasis subtype, health insurance scheme, geographical region, socioeconomic status and health service availability. Percentages are row percentages, calculated within each category of the corresponding factor. CI, confidence interval; CSMBS, Civil Servant Medical Benefit Scheme; Derm & device, dermatologist and phototherapy device available; OR, odds ratio; SSS, Social Security Scheme; UCS, Universal Coverage Scheme. *P < 0.05, **P < 0.01, ***P < 0.001.

The insurance scheme was strongly associated with access. Compared with UCS, patients under CSMBS had higher odds of access (aOR 1.88, 95% CI 1.52–2.34), whereas SSS beneficiaries had lower odds of access (aOR 0.33, 95% CI 0.26–0.43). Relative to Bangkok, access was significantly lower in the Central, Northeastern and Southern regions.

Patients in higher provincial income levels had greater odds of treatment, particularly those in level 4 (aOR 3.57, 95% CI 2.04–6.26) and level 5 (aOR 2.13, 95% CI 1.19–3.81). The availability of dermatologists and UV devices was independently associated with increased access (aOR 2.51, 95% CI 1.51–4.16).

### Interaction analysis

Interaction analysis showed that the association between health insurance schemes and phototherapy access varied across geographical regions (Table 3). The advantage associated with the CSMBS was observed in Bangkok (OR 1.68, 95% CI 1.18–2.37), the Central region (OR 2.56, 95% CI 1.63–4.05) and the Southern region (OR 3.66, 95% CI 1.96–6.84), but not in the Northern or Northeastern regions.

**Table 3:** Interaction between health insurance schemes and geographical regions associated with access to ultraviolet phototherapy

Region	CSMBS vs. UCS OR (95% CI)	SSS vs. UCS OR (95% CI)
Bangkok (reference)	1.68 (1.18–2.37)**	0.34 (0.23–0.50)***
Central	2.56 (1.63–4.05)***	0.16 (0.09–0.29)***
Northern	1.71 (0.93–3.14)	0.55 (0.30–1.00)*
Northeastern	1.20 (0.69–2.10)	0.15 (0.06–0.38)***
Southern	3.66 (1.96–6.84)***	1.37 (0.70–2.66)

Estimates were derived from a multilevel logistic regression model including an interaction term between health insurance schemes and geographical regions.

CI, confidence interval; CSMBS, Civil Servant Medical Benefit Scheme;OR, odds ratio; SSS, Social Security Scheme; UCS, Universal Coverage Scheme. ^*^P < 0.05, **P < 0.01, ***P < 0.001.

In contrast, beneficiaries under the SSS had significantly lower odds of receiving phototherapy in most regions, particularly in the Northeastern (OR 0.15, 95% CI 0.06–0.38) and Central regions (OR 0.16, 95% CI 0.09–0.29), while no significant association was observed in the Southern region and the effect in the Northern region was borderline significant.

### Inequality in phototherapy access

The Concentration Index for phototherapy utilization was 0.213, indicating income-related concentration in higher-income provinces (Figure 2). Similar patterns were observed for UV device availability, demonstrating clustering of resources in higher-income provinces.

**Figure 2:**
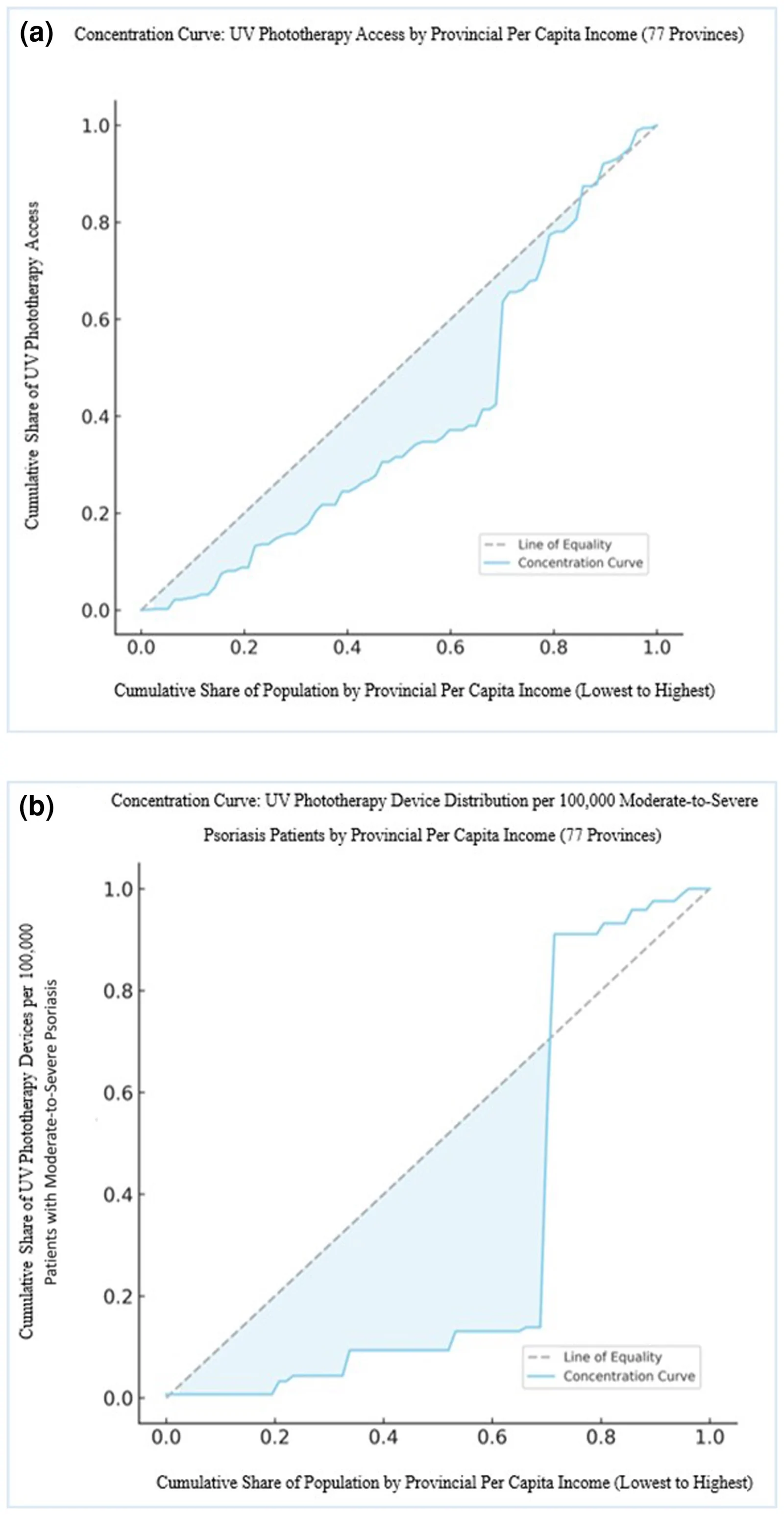
Concentration curves illustrating socioeconomic inequality in access to ultraviolet (UV) phototherapy in Thailand, 2023. (a) Access to UV phototherapy among patients with moderate-to-severe psoriasis. (b) Distribution of UV phototherapy devices per 100 000 patients with moderate-to-severe psoriasis. The x-axis represents the cumulative population ranked by provincial per capita income, and the *y*-axis represents the cumulative share of the outcome. Curves below the line of equality indicate pro-wealthy inequality.

## Discussion

This nationwide analysis indicates that access to UV phototherapy among patients with moderate-to-severe psoriasis in Thailand remains limited, with only 3.3% receiving treatment in 2023, despite universal health insurance coverage. Marked socio­economic and geographical inequalities in utilization were observed (Concentration Index 0.213), with treatment disproportionately concentrated in higher-income provinces.

Phototherapy utilization was significantly higher among beneficiaries of the CSMBS, individuals residing in higher-income ­provinces, and patients in regions with both dermatologists and available UV devices. In contrast, older patients (aged ≥50 years), individuals with nonplaque subtypes and residents outside Bangkok had lower odds of receiving treatment.

Importantly, the interaction analysis revealed that the effect of health insurance schemes on phototherapy access varied significantly across geographical regions. The advantage associated with the CSMBS was more pronounced in Bangkok, and Central and Southern regions, whereas disparities under the SSS were particularly evident in the Northeastern and Northern regions. These findings suggest that structural inequalities are not uniform, but context-­dependent, reflecting differences in regional health system capacity.

Taken together, these results indicate that demographic, clinical and health system factors collectively shape access to phototherapy, reflecting structural inequalities in service distribution within a universal health coverage context.^9,14–16^

The observed utilization rate may underestimate overall access, as private-sector services were not captured.

The observed patterns are consistent with Andersen’s Behavioral Model of Health Services Use,^12^ which emphasizes enabling resources, such as insurance, geographical accessibility and service availability, as key determinants of healthcare utilization. In this study, insurance scheme and regional service capacity appeared more strongly associated with phototherapy use than clinical characteristics alone.

Similarly, Penchansky and Thomas’s access framework^13^ ­highlights availability and geographical accessibility as central dimen­sions influencing care utilization. The concentration of dermatological resources in economically advantaged provinces may reflect broader gradients in healthcare infrastructure distribution.

International evidence demonstrates comparable trends. In the USA, socioeconomic disadvantage has been associated with reduced access to dermatological services,^17^ and rural populations often travel substantial distances for phototherapy,^8^ with services concentrated in tertiary centres.^18^ Metropolitan concentration of dermatological care has also been reported in Korea, despite universal coverage,^19^ while socio­economic variation in biologic access has been described across Asia.^20^ In Europe, lower-income groups report higher levels of unmet healthcare need.^21^

Collectively, these findings suggest that financial coverage alone does not fully address structural variation in specialist service access.^22,23^

The low utilization of phototherapy observed in this study probably reflects multiple barriers beyond financial coverage alone. Phototherapy requires frequent hospital visits, typically two to three times per week, which may impose substantial travel, time and indirect cost burdens, particularly for patients residing in underserved regions. In addition, uneven distribution of dermatological services, workforce limitations and administrative referral processes may further restrict access and continuity of care. Patient-level factors may also contribute, as some individuals may prefer more convenient systemic therapies or biologic agents over facility-based phototherapy, while others may have contra­indications that limit treatment suitability. Together, these ­findings suggest that both structural and patient-level factors influence phototherapy utilization within Thailand’s universal health coverage system.

The findings suggest that resource allocation should be prioritized towards underserved regions with a relatively high disease burden but limited service capacity, particularly the Northern and Northeastern regions. Strengthening phototherapy infrastructure and workforce availability in these areas is likely to reduce geographical and socioeconomic inequalities in access.

Given the facility-based nature of phototherapy, teledermatology-­supported triage and follow-up models can further enhance accessibility for patients residing in underserved areas. Aligning infrastructure development with regional epidemiological need is essential to achieving more balanced delivery of established dermatological therapies.

Alternative approaches, such as guided heliotherapy using controlled natural sunlight exposure, have been explored as potential strategies to improve access to ultraviolet-based treatment in resource-limited settings.^24,25^ Such approaches may be particularly relevant in settings where access to facility-based phototherapy is limited; however, further research is needed to evaluate their effectiveness, safety and feasibility within the Thai healthcare context.

This study integrates nationwide administrative insurance data with facility-level survey information, enabling a comprehensive assessment of patient-level and structural determinants of phototherapy utilization. The large national sample enhances the representativeness and generalizability of our findings within Thailand’s public healthcare system.

This study has several limitations. Firstly, phototherapy utilization was defined as at least one recorded session, which reflects initial access rather than treatment adequacy, continuity or adherence. Secondly, disease severity was defined using treatment-based proxies, which may introduce selection bias and ­confounding by indication, as treatment decisions may be influenced by factors not captured in the dataset. Thirdly, the exclusion of private-sector data may result in underestimation of both psoriasis prevalence and phototherapy utilization, as patients receiving care exclusively in private settings were not captured. Finally, detailed information on service capacity, including staffing, operational hours and treatment throughput, as well as patient-level factors such as preferences, contraindications, comorbidities and distance to care, were not available and may have influenced access.

Within Thailand’s universal health coverage system, meaningful socioeconomic and regional variation persists in access to UV phototherapy for psoriasis. Addressing structural differences in workforce distribution and service capacity may help promote more equitable access to evidence-based dermatological care in routine clinical practice.

## Data Availability

The data underlying this study were obtained from national administrative databases and are not publicly available due to institutional and ethical restrictions. Anonymized data may be available from the corresponding author upon reasonable request and with appropriate permissions. Administrative datasets derived from the National Health Security Office, Social Security Office and Central Office for Healthcare Information are not publicly available due to data privacy regulations and data-sharing agreements with respective national agencies.
